# Live Typhoid and Yellow Fever Vaccines Administered to a Patient With Ulcerative Colitis on Vedolizumab

**DOI:** 10.14309/crj.0000000000001507

**Published:** 2024-09-27

**Authors:** Yash Hegde, Mary S. Hayney, Freddy Caldera

**Affiliations:** 1 Department of Medicine, School of Medicine & Public Health, University of Wisconsin—Madison, Madison, WI; 2 School of Pharmacy, School of Medicine & Public Health, University of Wisconsin--Madison, Madison, WI; 3 Department of Medicine, Division of Gastroenterology and Hepatology, School of Medicine & Public Health, University of Wisconsin—Madison, Madison, WI

**Keywords:** vedolizumab, yellow fever vaccine, typhoid vaccine, inflammatory bowel disease

## Abstract

Patients with inflammatory bowel disease who receive immunosuppressive therapy have an increased risk of infection. Live vaccines are contraindicated in these patients because of the increased risk of unchecked replication of the attenuated vaccine microorganisms. Vedolizumab is a gut-selective biological agent with a low risk of infection approved for the treatment of inflammatory bowel disease. There are limited data on the risk of providing a live vaccine in patients receiving vedolizumab, and patients may receive live vaccines if the benefits outweigh the risks. We describe a patient with ulcerative colitis, treated with vedolizumab who received 2 live vaccines, typhoid and yellow fever, without postimmunization adverse events.

## INTRODUCTION

Patients with inflammatory bowel disease (IBD), including Crohn's disease (CD) and ulcerative colitis (UC), have heightened susceptibility to several infections.^1^ Advances in IBD treatment options include small molecule, anti-tumor necrosis factor agents, and non-tumor necrosis factor biologics, which have greater rates of clinical remission than conventional therapy but can result in increased risk of infections.^2^

Vedolizumab is a gut-selective anti-integrin (α4β7) monoclonal antibody used for the treatment of active CD and UC with a favorable safety profile.^3–5^ Although there are no data on the secondary transmission of infection by live vaccines in patients with IBD on vedolizumab, the US Food and Drug Administration label recommends that patients on the medication may receive live vaccines if the benefits outweigh the risks.^6^ The Centers for Disease Control and Prevention recommends patients on certain immunosuppressive therapy, including vedolizumab, not receive any live vaccines, although vedolizumab is not distinguished as gut-selective.^7^ We describe a patient with UC on vedolizumab that was vaccinated with oral live typhoid (Ty21a, trade name: Vivotif) and subcutaneous yellow fever (YF-17D, trade name: YF-VAX) vaccines.

## CASE REPORT

This is the case of a 25-year-old woman who was diagnosed with left-sided UC involving the rectum to distal transverse colon at the age of 15 years after recurrent episodes of diarrhea and hematochezia. Her condition was initially well managed with aminosalicylates until she developed worsening symptoms of fecal urgency and hematochezia with a repeat colonoscopy demonstrating active inflammation. Vedolizumab was initiated and maintained at 300 mg every 8 weeks, and she achieved clinical and endoscopic remission with no active disease in interval periods.

Fifty months after the initiation of vedolizumab and continued clinical remission, she planned to travel to Brazil and was prescribed 2 live vaccines at an outside facility: the oral Ty21a vaccine and subcutaneous YF-17D vaccine. We reviewed the risks and benefits of providing live vaccines to patients receiving vedolizumab and counseled her regarding the lack of evidence concerning the safety of live vaccines administered to these patients. The patient decided to receive these 2 live vaccines within the same week, 4 days before the next scheduled dose of vedolizumab. Her next dose of vedolizumab was consequently held. She traveled to Brazil 10 days later for a month.

Ten days after returning from the trip, she visited our clinic for a follow-up appointment. She tolerated both live vaccines without adverse reactions and reported no fever, rash, jaundice, malaise, or myalgia. All laboratory test results were normal, including complete blood count, basic metabolic panel, and aspartate transaminase and alanine transaminase levels. She resumed vedolizumab infusions and maintained clinical remission with no development of UC symptoms during and after travel.

## DISCUSSION

Patients with IBD should receive inactivated vaccines, but those on immunosuppressive therapy are not recommended to receive live vaccines due to the concern of infection with attenuated virus or bacteria.^8^ This is the first reported case of a patient receiving vedolizumab who received live Ty21a and YF-17D vaccines.

The literature on live vaccine administration in patients receiving vedolizumab is limited. Wichmann et al described a patient with CD on vedolizumab and methotrexate that received a live measles, mumps, and rubella (MMR) vaccine with no adverse events after vaccination.^9^ In their prospective cohort study, Shiga et al administered live MMR or varicella vaccines to patients with IBD with negative serologic titers and without a verified immunization history on vedolizumab monotherapy with a high rate of seroprotection (92%) and no complications, including vaccine-induced infections, after immunization.^10^ These reports are limited because they might have boosted immunity in individuals who were already immune. In the first report, the patient had been previously immunized with 2 doses of the MMR vaccine and received a live MMR vaccine due to a low serologic titer to measles. Similarly, the case series immunized individuals without a verified immunization history and a low titer of measles, mumps, rubella, or varicella. The Advisory Committee on Immunization Practice does not recommend using serological titers to evaluate immunity to MMR or varicella in patients with a previous immunization history.^11,12^ Therefore, these patients were potentially immune to MMR or varicella and received a vaccine to boost their immunity and not induce primary immunity. Thus, the evaluation of the safety of MMR or varicella live vaccines in patients receiving vedolizumab is limited.

Our case is different because both YF-17D and Ty21a vaccines induce primary immunity because individuals are not routinely vaccinated or immune to these diseases. Data on the administration of the YF-17D vaccine in patients with IBD on immunosuppressive therapy are limited, and these patients remain at high risk of infection when traveling to endemic areas in South America and Africa. Rüddel et al presented a patient with UC on infliximab who received the YF-17D vaccine with a postvaccination course notable for a fever and elevated liver enzymes that ultimately resolved with no further clinical complications.^13^ Ekenberg et al described a patient with CD on infliximab who received the YF-17D vaccine with postimmunization complications significant for a short duration of fever, headache, generalized weakness, elevated liver enzymes, and yellow fever viremia.^14^ The patient's symptoms resolved. In our patient, administration of the YF-17D vaccine yielded no postvaccination complications suggesting this live vaccine may be safe in patients on vedolizumab.

Very limited data are available in patients with IBD who received live Ty21a vaccines. A systematic review of live vaccines in immunosuppressed patients reported 10 individuals with immune-mediated inflammatory diseases, including IBD, who received a live Ty21a vaccine with no reports of local or systemic adverse events or infection with the vaccine strain.^15^

The literature on the timing of live vaccine use in patients on biologic therapy remains limited as all live vaccine use in these patients is contraindicated. It is known that inactivated vaccines can be administered at any time, and timing of biologic therapy does not impact vaccine response.^16^

Limited data on live vaccine administration in patients with IBD receiving vedolizumab demonstrate no adverse events postvaccination. Future studies are needed evaluating the safety of live vaccine in patients on vedolizumab and other immune modifying therapies.

## DISCLOSURES

Author contributions: Y. Hegde: drafting of case report and critical revision of case report. MS Hayney: critical revision of case report. F. Caldera: drafting of case report and critical revision of case report, and is the article guarantor.

Financial disclosure: Dr Freddy Caldera has received research support from GSK, Takeda Pharmaceuticals, Janssen and Novavax. He has been a consultant for Takeda, Arena Pharmaceuticals, GSK, and Celgene. Dr Mary Hayney is a consultant for GSK Vaccines and has received research support from Takeda Pharmaceuticals and Dynavax.

Informed consent was obtained for this case report.
